# The influence of clinical coaching teams on quality of entrustable professional activity assessments

**DOI:** 10.1002/aet2.10879

**Published:** 2023-06-23

**Authors:** Simranjeet Singh, Warren J. Cheung, Sebastian Dewhirst, Timothy J. Wood, Jeffrey M. Landreville

**Affiliations:** ^1^ University of Ottawa Ottawa Ontario Canada; ^2^ Department of Emergency Medicine University of Ottawa Ottawa Ontario Canada; ^3^ Royal College of Physicians and Surgeons of Canada Ottawa Ontario Canada; ^4^ Department of Innovation in Medical Education University of Ottawa Ottawa Ontario Canada

## Abstract

**Background:**

Coaching is an important component of workplace‐based assessment in competency‐based medical education. Longitudinal coaching relationships have been proposed to enhance the trainee–supervisor relationship and promote high‐quality assessment.

**Objective:**

The objective of this study was to determine the influence of longitudinal coaching relationships on the quality of entrustable professional activity (EPA) assessments.

**Methods:**

EPAs (*n* = 174) completed by emergency medicine (EM) supervisors between July 2020 and June 2021 were extracted and divided into two groups; one group consisted of EPAs completed by supervisors when a longitudinal coaching relationship existed (*n* = 87) and the other group consisted of EPAs completed by the same supervisors when no coaching relationship existed (*n* = 87). Three physicians were recruited to rate the EPAs using the Quality of Assessment and Learning (QuAL) score, a previously published measure of EPA quality. An analysis of variance was performed to compare mean QuAL scores between the groups. Linear regression analysis was conducted to examine the relationship between trainee performance (EPA rating) and EPA assessment quality (QuAL score).

**Results:**

All raters completed the survey. The mean ± SD QuAL score in the coaching relationship group (3.63 ± 0.91) was higher than the no coaching relationship group (3.51 ± 1.10) but the difference was not statistically significant (*p* = 0.40). Supervisor was a significant predictor of QuAL score (*p* = 0.012) and supervisor alone accounted for 26% of the variability in QuAL scores (R^2^ = 0.26). There was no significant relationship between trainee performance and EPA assessment quality.

**Conclusions:**

The presence of a longitudinal coaching relationship did not influence the quality of EPA assessments.

## INTRODUCTION

The role of clinical supervisors in competency‐based medical education (CBME) has evolved to include a focus on observation of trainees in the workplace.^1^ These observations provide an opportunity to move beyond traditional approaches to feedback and engage in coaching.^2^

Historically, feedback to trainees has been of poor quality and low reliability and often does not lead to actionable suggestions for performance improvement.^3^, ^4^, ^5^, ^6^, ^7^, ^8^ Episodic, fragmented, and limited interactions between supervisor and trainee act as barriers to meaningful assessments.^9^ The unwillingness of supervisors to provide negative feedback to trainees with whom they have limited familiarity has led to the development of politeness strategies and the “failure to fail” phenomenon to smooth social functioning.^4^, ^10^, ^11^

Longitudinal coaching in medical education has been introduced to enhance the trainee–supervisor relationship, promote continuous feedback, and improve clinical performance.^12^ Increased time spent with trainees has previously been shown to help supervisors provide more specific and individualized feedback, witness performance improvement as a result of feedback exchange, and identify trainees in difficulty.^13^, ^14^, ^15^, ^16^, ^17^, ^18^, ^19^, ^20^ However, the impact of longitudinal coaching initiatives on the quality of entrustable professional activity (EPA) assessments has not been studied.^21^

Workplace‐based assessments (WBA) such as EPAs are critical for trainees to fuel growth as well as for residency program leaders to make decisions pertaining to trainee promotion and readiness for independent practice. The resource intensive nature of coaching and the reliance on EPAs for trainee progression in CBME warrants an investigation into the value of such relationships.^22^ The objective of this study was to evaluate the impact of supervisor–trainee continuity in the form of longitudinal clinical coaching teams (CCT) on the quality of EPA assessments.

## METHODS

### Setting and population

This study was conducted in the department of emergency medicine (DEM) at the University of Ottawa, Canada, in 2021.^23^, ^24^ Trainees in their second year and above within the DEM's CBME EM residency program are assigned to CCTs comprising of one EM trainee and one supervising physician (clinical coach). They work in a 1:1 ratio and are scheduled to work matched shifts together (typically 4–6 per month) for a specified period of time (typically 2–3 months). All clinical teaching faculty within the DEM are eligible to be assigned as clinical coaches for a CCT pairing. CCTs pairings are assigned by the residency program director taking into consideration the trainees individual learning needs and goals.

### Design

EPAs from trainees (*n* = 20) during the study period (July 2020–June 2021) were extracted, deidentified, and segregated based on completion following a CCT or non‐CCT (NCCT) interaction in the emergency department. During the study period, 29 teaching faculty participated in CCTs. For each supervisor, six assessments (three CCT and three NCCT) were randomly selected. Supervisors with less than three CCT or NCCT EPAs were excluded to maintain an equal number of assessments from all supervisors.

An online survey was created to present these EPAs in a randomized order. Three EM physician raters were recruited to rate the quality of the assessment documented on the selected EPA forms using the QuAL score. A previous study has shown that the QuAL score requires three raters to reach an acceptable target reliability of 0.79.^25^

### Outcome measure

The QuAL score was used to objectively rate the quality of short, workplace‐based comments contained within EPA assessments.^26^ A QuAL score of 5 represents the highest possible score whereas a score of 0 is the lowest possible score. Validity evidence for the QuAL score has been demonstrated for short WBA such as EPAs, including high reliability and strong correlation with rater perception of utility.^27^

### Data analysis

A comparison of mean QuAL scores between the CTT and NCCT study groups was conducted. A factorial analysis of variance (ANOVA) was conducted with mean QuAL score as the dependent variable and presence of a CCT relationship as the independent variable. ANOVA analysis with only supervisor as an independent variable was also performed for comparison. The relationship between the trainee performance (given by the EPA entrustment scale rating) and QuAL score was examined using a linear regression analysis with EPA entrustment scale rating as the independent variable and QuAL score as the dependent variable. Separate linear regression analyses were conducted for both the CCT and the NCCT groups.

### Ethics statement

This study received ethics exemption from the Ottawa Health Science Network Research Ethics Board.

## RESULTS

A total of 174 EPAs completed by 29 supervisors (CCT 87, NCCT 87) were rated using the QuAL score by all raters (*n* = 3). Of the EPAs rated, 33 supervisors contributed EPA assessments for 20 trainees. The mean ± SD number of CCT shifts per resident was 15.8 ± 5.3 (range 8–34). The difference in mean ± SD QuAL score between the CCT group (3.63 ± 0.91) and NCCT group (3.51 ± 1.04) was not statistically significant (*p* = 0.40).

Supervisor alone accounted for 26% of the variability in QuAL scores (F = 1.82, *p* = 0.012, R^2^ = 0.26). Adding in the presence of CCT to supervisor did not explain any additional variability in QuAL score compared to supervisor alone. There was no relationship between trainee performance (EPA entrustment scale rating) and EPA assessment quality (QuAL score) (*p* = 0.93).

## DISCUSSION

Our study did not demonstrate any significant impact of supervisor–trainee continuity in the form of longitudinal CCTs on the quality of EPA assessments. The results align with those of a similar study by Cheung et al.^9^ performed in a non‐CBME curriculum. EPA assessments only represent the documented aspect of feedback and do not encompass the verbal feedback that occurs in the moment. However, competence committees are not privy to verbal feedback and rely on documented EPA data to make high‐stakes progression decisions.

## LIMITATIONS

Although some studies have demonstrated the superiority of feedback obtained during longitudinal supervisor–trainee relationships, their results are derived from qualitative analysis based on the perception of assessments by supervisors and trainees.^14^, ^15^, ^17^, ^18^, ^19^, ^20^ This is perhaps a more effective model to assess the role of continuity on resident *learning* rather than documented assessments. Furthermore, we do recognize that the longitudinal CCT model is unique to our institution and other programs may have implemented forms of enhanced supervisor–learner continuity differently, limiting the generalizability of our findings.

Scheduling challenges make standardization of continuity difficult. It is possible that certain randomly selected EPAs were from early interactions between trainee and supervisor. However, Telio et al.^28^ demonstrated that residents judged their educational alliance by the supervisor's engagement as an educator rather than the amount of time spent together. The complexity of feedback encompasses the role of learner culture, perceptions of credibility, and trainee receptivity to feedback which may not be mitigated by increasing continuity.^29^

In this study, supervisors were more significant predictors of the quality of EPA assessment than supervisor–trainee continuity. There remains heterogeneity in the capability of individual supervisors to document high‐quality assessments of residents.^30^ Faculty development has been shown to modestly improve the quality of written feedback and additional faculty development related to EPA assessment documentation may be of benefit.^7^, ^30^, ^31^, ^32^ Although an inverse relationship between quality of assessment and trainee performance has previously been observed in non‐CBME systems,^3^, ^9^ our study failed to replicate this finding. One hypothesis is that EPAs are more task oriented compared to other forms of WBA and may lend itself to higher quality assessment regardless of trainee performance. Future studies involving a qualitative analysis of trainee and supervisor perspectives on longitudinal coaching relationships may offer a deeper understanding of the perceived benefits and challenges of coaching in medical education as well as its role in learning and assessment.

## CONCLUSIONS

Increased supervisor–trainee continuity in the form of longitudinal clinical coaching teams did not demonstrate a significant improvement in the quality of entrustable professional activity assessments.

## FUNDING INFORMATION

This study was funded by an internal research grant from the Department of Emergency Medicine, University of Ottawa.

## CONFLICT OF INTEREST STATEMENT

The authors declare no conflicts of interest.

## References

[1] Gofton W , Dudek N , Barton G , Bhanji F . Workplace‐Based Assessment Implementation Guide: Formative Tips for Medical Teaching Practice. 1st ed. The Royal College of Physicians and Surgeons of Canada; 2017:1‐12.

[2] Landreville J , Cheung W , Frank J , Richardson D . A definition for coaching in medical education. Can Med Educ J. 2019;10(4):e109‐e110. 31814864 PMC6892322

[3] Jackson JL , Kay C , Jackson WC , Frank M . The quality of written feedback by attendings of internal medicine residents. J Gen Intern Med. 2015;30(7):973‐978. 25691242 10.1007/s11606-015-3237-2 PMC4471022

[4] Ginsburg S , van der Vleuten C , Eva KW , Lingard L . Hedging to save face: a linguistic analysis of written comments on in‐training evaluation reports. Adv Health Sci Educ Theory Pract. 2016;21(1):175‐188. 26184115 10.1007/s10459-015-9622-0

[5] Scarff CE , Bearman M , Chiavaroli N , Trumble S . Keeping mum in clinical supervision: private thoughts and public judgements. Med Educ. 2019;53(2):133‐142. 30328138 10.1111/medu.13728

[6] Anderson PAM . Giving feedback on clinical skills: are we starving our young? J Grad Med Educ. 2012;4(2):154‐158. 23730434 10.4300/JGME-D-11-000295.1 PMC3399605

[7] Berbano EP , Browning R , Pangaro L , Jackson JL . The impact of the Stanford faculty development program on ambulatory teaching behavior. J Gen Intern Med. 2006;21(5):430‐434. 16704383 10.1111/j.1525-1497.2006.00422.x PMC1484783

[8] Moss HA , Derman PB , Clement RC . Medical student perspective: working toward specific and actionable clinical clerkship feedback. Med Teach. 2012;34(8):665‐667. 22830325 10.3109/0142159X.2012.687849

[9] Cheung WJ , Dudek NL , Wood TJ , Frank JR . Supervisor‐trainee continuity and the quality of work‐based assessments. Med Educ. 2017;51(12):1260‐1268. 28971502 10.1111/medu.13415

[10] Dudek NL , Marks MB , Regehr G . Failure to fail: the perspectives of clinical supervisors. Acad Med J Assoc Am Med Coll. 2005;80(10 Suppl):S84‐S87. 10.1097/00001888-200510001-00023 16199466

[11] Kilbertus S , Pardhan K , Zaheer J , Bandiera G . Transition to practice: evaluating the need for formal training in supervision and assessment among senior emergency medicine residents and new to practice emergency physicians. CJEM. 2019;21(3):418‐426. 30841941 10.1017/cem.2019.8

[12] Brown LE , Rangachari D , Melia M . Beyond the Sandwich: from feedback to clinical coaching for residents as teachers. MedEdPORTAL J Teach Learn Resour. 2017;13:10627. 10.15766/mep_2374-8265.10627 PMC6354721 30800828

[13] Delva D , Sargeant J , Miller S , et al. Encouraging residents to seek feedback. Med Teach. 2013;35(12):e1625‐e1631. 23848343 10.3109/0142159X.2013.806791

[14] Rassbach CE , Blankenburg R . A novel pediatric residency coaching program: outcomes after one year. Acad Med. 2018;93(3):430‐434. 28700460 10.1097/ACM.0000000000001825

[15] Mazotti L , O'Brien B , Tong L , Hauer KE . Perceptions of evaluation in longitudinal versus traditional clerkships. Med Educ. 2011;45(5):464‐470. 21486322 10.1111/j.1365-2923.2010.03904.x

[16] Boscardin CK , Wijnen‐Meijer M , Cate OT . Taking rater exposure to trainees into account when explaining rater variability. J Grad Med Educ. 2016;8(5):726‐730. 28018538 10.4300/JGME-D-16-00122.1 PMC5180528

[17] Ogur B , Hirsh D , Krupat E , Bor D . The Harvard Medical School‐Cambridge integrated clerkship: an innovative model of clinical education. Acad Med J Assoc Am Med Coll. 2007;82(4):397‐404. 10.1097/ACM.0b013e31803338f0 17414198

[18] Michelson CD , Dzara K , Ramani S , Vinci R , Schumacher D . Keystone: exploring pediatric residents' experiences in a longitudinal integrated block. Teach Learn Med. 2019;31(1):99‐108. 30303403 10.1080/10401334.2018.1478732

[19] Tannenbaum DW . New “horizontal” curriculum in family medicine residency. Can Fam Physician Med Fam Can. 1998;44:1669‐1675. PMC2277755 9721423

[20] Playford D , Kirke A , Maley M , Worthington R . Longitudinal assessment in an undergraduate longitudinal integrated clerkship: the mini clinical evaluation exercise (mCEX) profile. Med Teach. 2013;35(8):e1416‐e1421. 23544917 10.3109/0142159X.2013.778392

[21] Hirsh DA , Ogur B , Thibault GE , Cox M . “Continuity” as an organizing principle for clinical education reform. N Engl J Med. 2007;356(8):858‐866. 17314348 10.1056/NEJMsb061660

[22] Lee AS , Ross S . Continuity of supervision: does it mean what we think it means? Med Educ. 2021;55(4):448‐454. 32929800 10.1111/medu.14378

[23] Rekman J , Gofton W , Dudek N , Gofton T , Hamstra SJ . Entrustability scales: outlining their usefulness for competency‐based clinical assessment. Acad Med J Assoc Am Med Coll. 2016;91(2):186‐190. 10.1097/ACM.0000000000001045 26630609

[24] Tavares W , Gofton W , Bhanji F , Dudek N . Reframing the O‐SCORE as a retrospective supervision scale using validity theory. J Grad Med Educ. 2022;14(1):22‐24. 35222815 10.4300/JGME-D-21-00592.1 PMC8848889

[25] Landreville JM , Wood TJ , Frank JR , Cheung WJ . Does direct observation influence the quality of workplace‐based assessment documentation? AEM Educ Train. 2022;6(4):e10781. 35903424 10.1002/aet2.10781 PMC9305723

[26] Chan TM , Sebok‐Syer SS , Sampson C , Monteiro S . The quality of assessment of learning (Qual) score: validity evidence for a scoring system aimed at rating short, workplace‐based comments on trainee performance. Teach Learn Med. 2020;32(3):319‐329. 32013584 10.1080/10401334.2019.1708365

[27] Woods R , Singh S , Thoma B , et al. Validity evidence for the quality of assessment for learning score: a quality metric for supervisor comments in competency based medical education. Can Med Educ J. 2022;13(6):19‐35. Available from: https://journalhosting.ucalgary.ca/index.php/cmej/article/view/74860 36440075 10.36834/cmej.74860 PMC9684040

[28] Telio S , Regehr G , Ajjawi R . Feedback and the educational alliance: examining credibility judgements and their consequences. Med Educ. 2016;50(9):933‐942. 27562893 10.1111/medu.13063

[29] Ramani S , Könings KD , Ginsburg S , van der Vleuten CPM . Twelve tips to promote a feedback culture with a growth mind‐set: swinging the feedback pendulum from recipes to relationships. Med Teach. 2019;41(6):625‐631. 29411668 10.1080/0142159X.2018.1432850

[30] Kogan JR , Conforti LN , Bernabeo EC , Durning SJ , Hauer KE , Holmboe ES . Faculty staff perceptions of feedback to residents after direct observation of clinical skills: faculty staff perceptions about feedback after observation. Med Educ. 2012;46(2):201‐215. 22239334 10.1111/j.1365-2923.2011.04137.x

[31] Holmboe ES , Fiebach NH , Galaty LA , Huot S . Effectiveness of a focused educational intervention on resident evaluations from faculty a randomized controlled trial. J Gen Intern Med. 2001;16(7):427‐434. 11520379 10.1046/j.1525-1497.2001.016007427.x PMC1495246

[32] Salerno SM , O'Malley PG , Pangaro LN , Wheeler GA , Moores LK , Jackson JL . Faculty development seminars based on the one‐minute preceptor improve feedback in the ambulatory setting. J Gen Intern Med. 2002;17(10):779‐787. 12390554 10.1046/j.1525-1497.2002.11233.x PMC1495113

